# Individual differences in working memory capacity and cue-guided behavior in humans

**DOI:** 10.1038/s41598-019-43860-w

**Published:** 2019-05-13

**Authors:** Sara Garofalo, Simone Battaglia, Giuseppe di Pellegrino

**Affiliations:** Centre for Studies and Research in Cognitive Neuroscience, Department of Psychology, University of Bologna, Bologna, Italy

**Keywords:** Decision, Learning and memory

## Abstract

Information gathered via Pavlovian and Instrumental learning can be integrated to guide behavior, in a phenomenon experimentally known as Pavlovian-to-Instrumental Transfer (PIT). In particular, in appetitive PIT, a reward-associated cue is able to enhance the instrumental response previously associated with the same (outcome-specific PIT), or a similar (general PIT), reward. The PIT effect is increasingly investigated for its numerous implications in clinical contexts as well as daily life situations. Nevertheless, the precise mechanism behind it is not yet clear. The relation between the PIT effect and high-level cognitive abilities - like working memory - is still unknown, but potentially relevant to unveil its functioning. The present study aims to examine the precise relationship between individual differences in working memory and the two forms of PIT effect, namely outcome-specific and general. For this purpose, 100 participants underwent a classical PIT paradigm. Results showed a relationship between individual working memory and outcome-specific PIT, but not general PIT. Importantly, the role of working memory was not related to the acquisition of the learning contingencies, but rather linked to an imbalance between congruent and incongruent choices. The results are discussed in terms of the adaptive and maladaptive implications for human behavior.

## Introduction

In daily life, we learn which actions and which environmental circumstances can lead us to achieve a goal. Two very basic learning mechanisms involved in this ability are the well-known instrumental and Pavlovian conditioning^1^. With instrumental conditioning, we learn to predict the consequences of a given action, whereas with Pavlovian conditioning we learn to predict the consequences associated with the presence of external cues. Information gathered via such learning processes, even if independently acquired, can be integrated to guide behaviour^2–4^. An example of this mechanism can be observed in the Pavlovian-to-Instrumental Transfer (PIT) effect, which tests the extent to which a Pavlovian stimulus (S) can drive instrumental responses (R) independently paired with the same or a similar outcome (O).

According to some authors, instrumental responses can be controlled by either an R-O association (e.g., pressing the button is rewarded with chocolate), or by an O-R association (e.g., I want chocolate, so I press the button)^5,6^. And it is also acknowledged in the literature that the presentation of a Pavlovian stimulus can activate both a general motivational representation of the potential reward value (i.e., food craving), and a representation of the specific sensory features of the precise outcome at stake (e.g., dark chocolate)^7^. So, how can these two mechanisms interact? The cross connection between the two paths to response control, and the two representations that a Pavlovian stimulus can elicit, is at the foundation of two separate forms of transfer, namely outcome-specific PIT and general PIT^2,3,7,8^. In outcome-specific PIT, the Pavlovian stimulus exerts a selective influence on instrumental responses associated with the same reinforcer via a more^5,9^ or less^10^ direct link to the outcome representation. That is, a Pavlovian stimulus associated with dark chocolate will trigger responses specifically associated with dark chocolate. Conversely, in general PIT the stimulus invigorates instrumental responses paired with motivationally similar reinforcers (i.e., reinforces with the same value) which is detached by the specific sensory properties of the outcome^2,3^. For example, a Pavlovian stimulus associated with dark chocolate triggers responses associated with any kind of food.

Such interaction between instrumental and Pavlovian learning systems determines a cue-guided behaviour, which can present both adaptive and maladaptive implications. It can trigger a flexible response that facilitates the achievement of a goal (e.g., obtain food when hungry), but it can also lead to an inflexible association when a response is perseverated in absence of a reward or despite a negative consequence^3,11^. Most literature focused on the latter implication. In fact, the PIT effect has been associated with many clinical conditions, such as addiction^12–15^, compulsive behavior^16^ and other neuropsychiatric disorders, like depression^17^ or schizophrenia^18^. Previous findings^11^ suggested that individual differences in learning style may play a role in the predisposition to such maladaptive implications. A PIT-like effect can also be observed in marketing strategies, where a logo or a jingle can prime a potential consumer to purchase the associated product^19^.

Given the repercussions in clinical contexts and daily life, the PIT effect is increasingly investigated in humans and has been largely examined also in non-human animals^2,3^. However, the precise mechanism behind this effect is not yet clear and whether the influence that a Pavlovian stimulus can exert on instrumental choices can be attributed to more strategic cognitive components, or to more automatic determinants, is still hotly debated^12,20–22^.

In particular, the relation between the PIT effect and high-level cognitive abilities – such as working memory - is still unknown, but potentially relevant to shed new light on this effect. A number of findings suggest that working memory is crucially involved in decision-making, especially when the ability to integrate past experiences about action and outcome values is needed to guide current decisions^18,23,24^. By definition, working memory is the ability to maintain information so that it can be integrated with other information and used to guide behavior; it is involved in maintaining current goals by selecting relevant information and inhibiting irrelevant stimuli^25,26^. Crucially, the PIT effect requires the combination of different pieces of information without re-exposure to the outcome, a process which is very likely to require working memory^27^. When facing multiple R− > O (instrumental learning) and S− > O (Pavlovian learning) associations, individual differences in working memory may, thus, influence the accessibility of such representations to control subsequent behavioral expressions^28^. Interestingly, recent findings suggest that high working memory capacity is not associated with the expression of conditioning *per se* (i.e., learning of the contingencies), but rather with a more effective use of the acquired information in later phases^29^. In particular, as compared to general PIT, outcome-specific PIT requires retaining a detailed representation of the outcomes available and of each response-outcome pairing^30^, thus it should rely more on working memory.

**Figure 1:**
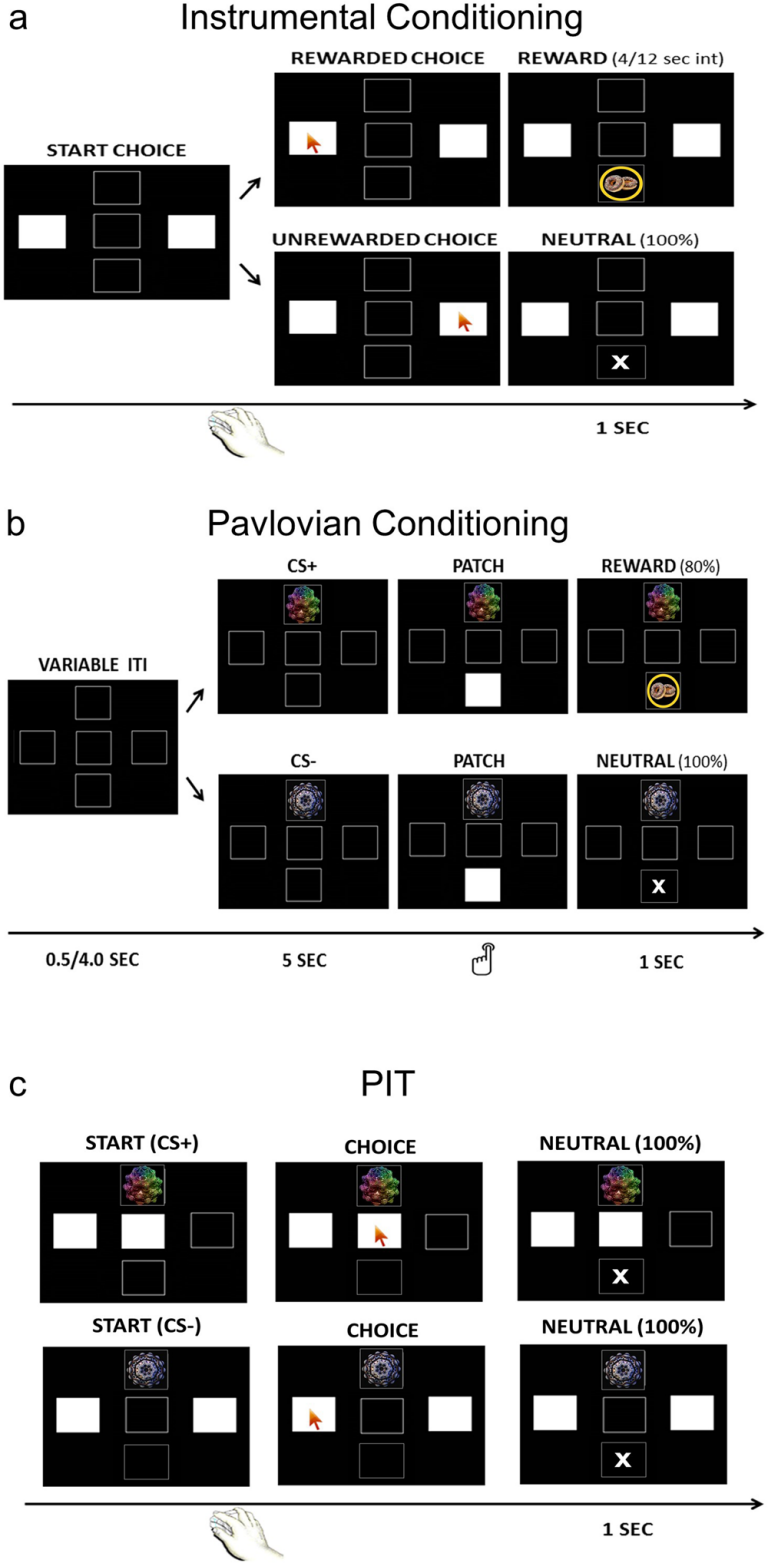
Pavlovian-to-Instrumental Transfer (PIT) task. Visual representation of the three task phases to test for the PIT effect. During instrumental conditioning (**a**) participants learned the association between three instrumental responses and the associated reward or neutral outcome. During Pavlovian conditioning (**b**) the same rewards were associated with visual stimuli. During PIT (**c**) participants were asked to perform the again an instrumental choice under extinction (i.e., in the absence of rewards) while being presented with task-irrelevant Pavlovian stimuli.

Based on these premises, the present experiment aims to test to which extent outcome-specific and general PIT effect are related to individual working memory capacity. For this purpose, 60 participants performed a PIT task (Table 1), consisting of three phases: instrumental conditioning, in which they learned the association between three instrumental responses and the relative reward or neutral outcome (Fig. 1a); Pavlovian conditioning, in which the same rewards were associated with visual stimuli (Fig. 1b); PIT, in which the participants were asked to perform an instrumental choice under extinction (i.e., in absence of rewards) while being presented with task-irrelevant Pavlovian stimuli (Fig. 1c). The aim of this last phase is to test the ability of Pavlovian stimuli to promote instrumental responses previously associated with a reward (i.e., PIT effect). Working memory ability was tested with the Automated Operation Span Task^31^.

The hypothesis is that individual differences in working memory will be reflected in the individual tendency to show either outcome-specific or general PIT. More precisely, supporting a detailed representation of the associations between each response and their outcomes, higher working memory capacity should be associated with larger outcome-specific PIT effect. Conversely, working memory should play a more marginal role when the transfer effect reflects enhancement of responding by a motivational component, like in the case of general PIT. Furthermore, the stage in which working memory is more relevant will be analyzed by relating individual working memory ability not only to the PIT effect itself but also to the strength of instrumental and Pavlovian learning.

## Results

### Instrumental conditioning

#### Implicit learning

**Figure 2:**
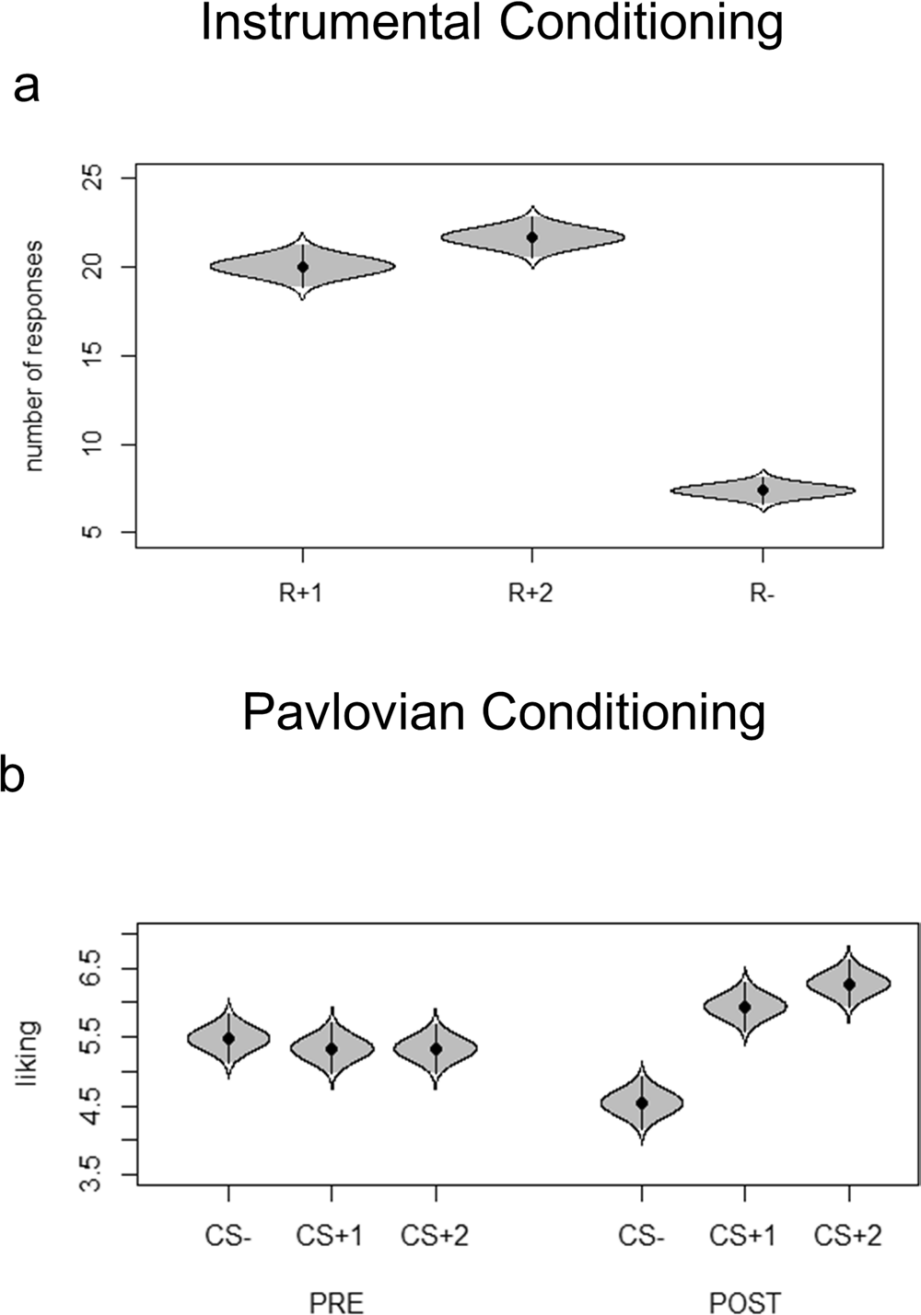
Cat’s eye plot of instrumental and Pavlovian learning. Panel (a) shows the number of responses performed during the instrumental conditioning, where a significant difference between reward-associated responses (R+1, R+2) and unrewarded response (R−) was found. Panel (b) shows the liking scores of the stimuli before and after undergoing Pavlovian conditioning, where an was observed increase for the reward-associated stimuli (CS+1, CS+2) and a reduction for the unrewarded stimulus (CS−). Overall, these results confirm that participants correctly learned instrumental and Pavlovian contingencies.

To ensure learning of instrumental contingencies, the number of rewarded (R+1, R+2) and unrewarded (R−) responses (mouse clicks) performed during each 10-second trial was compared. Response (R+1/R+2/R−) served as the independent variable and the average number of responses performed as the dependent variable. Results showed a main effect of response (F(1.46, 144.38) = 192.75; p < 0.0001; part. η^2^ = 0.66; BF_10_ = 2.39e + 72; err% = 1.53). Post-hoc analysis confirmed a statistically significant difference (ps <0.0001) between R + 1 (number of responses m = 20.05; sd = 8.54) and R− (number of responses m = 7.38; sd = 5.46), as well as between R+2 (number of responses m = 21.68; sd = 8.04) and R−. Figure 2a shows means and clearly separate 95% confidence intervals. These results indicate a preference for both reward-paired responses (R+1 and R+2) as compared to the unrewarded response (R−), thus indicating successful instrumental learning. Critically, no preference between the two rewarded responses emerged.

#### Explicit learning

Analysis of explicit associations confirmed that participants correctly reported the response-outcome associations on 93.33% of cases.

### Pavlovian conditioning

To ensure Pavlovian learning, two measures were considered: reaction times to the presentation of the patch preceding the outcome, and the pre-post change in liking of the conditioned stimuli (CSs).

#### Implicit learning (CS liking)

Phase (pre/post conditioning) and CS (CS+1/CS+2/CS−) served as independent variables. Liking scores served as dependent variable. Results showed a significant phase by CS interaction (F(2.196) = 44.319; p < 0.001; part. η^2^ = 0.32; BF_10_ = 1706255; err% = 1.47). Post-hoc analysis showed a significant difference between CS+1 pre (m = 5.33; sd = 1.87) and CS+1 post (m = 5.94; sd = 1.77; p = 0.001), between CS+2 pre (m = 5.34; sd = 1.81) and CS+2 post (m = 6.30; sd = 1.75; p < 0.001), and between CS− pre (m = 5.47; sd = 1.81) and CS− post (m = 4.51; sd = 1.91; p < 0.001). Importantly: in the pre-conditioning phase, there were not differences in liking scores between all CSs (p = 1); in the post-conditioning phase, liking scores between CSs+ were significantly different from CS− (p < 0.001) but not between CS+1 and CS+2 (p = 0.28). Overall, these results confirm that Pavlovian conditioning did occur. More specifically they show that (1) before Pavlovian training all stimuli were equally liked, and that (2) after Pavlovian training the liking for both CS+1 and CS+2 increased while the liking for CS− decreased (Fig. 2b). This last evidence might be explained by the relative devaluation of the CS− when compared to the reward-associated stimuli in the post-conditioning phase.

#### Implicit learning (reaction times)

CS (CS+1/CS+2/CS−) served as the independent variable and reaction times as the dependent variable. Results showed a significant main effect of CS (F(1.34, 132.98) = 10.30; p = 0.0005; part. η^2^ = 0.09; BF_10_ = 1.82e + 16; err% = 0.61). Post-hoc analysis confirmed a statistically significant difference (ps < 0.006) between CS+1 (milliseconds m = 302.74; sd = 74.84) and CS− (milliseconds m = 318.95; sd = 88.01), as well as between CS+2 (milliseconds m = 295.84; sd = 67.99) and CS−, but not between CS+1 and CS+2 (p = 0.1). Participants were significantly faster when a reward was anticipated (CS+1, CS+2) as compared to when no reward was expected (CS−). This reward-specific response facilitation confirms successful Pavlovian conditioning^11^.

#### Explicit learning

Analysis of explicit associations confirmed that participants correctly reported the stimulus-outcome associations on 96.11% of cases.

### Pavlovian-to-Instrumental Transfer (PIT)

#### Outcome-specific PIT

**Figure 3:**
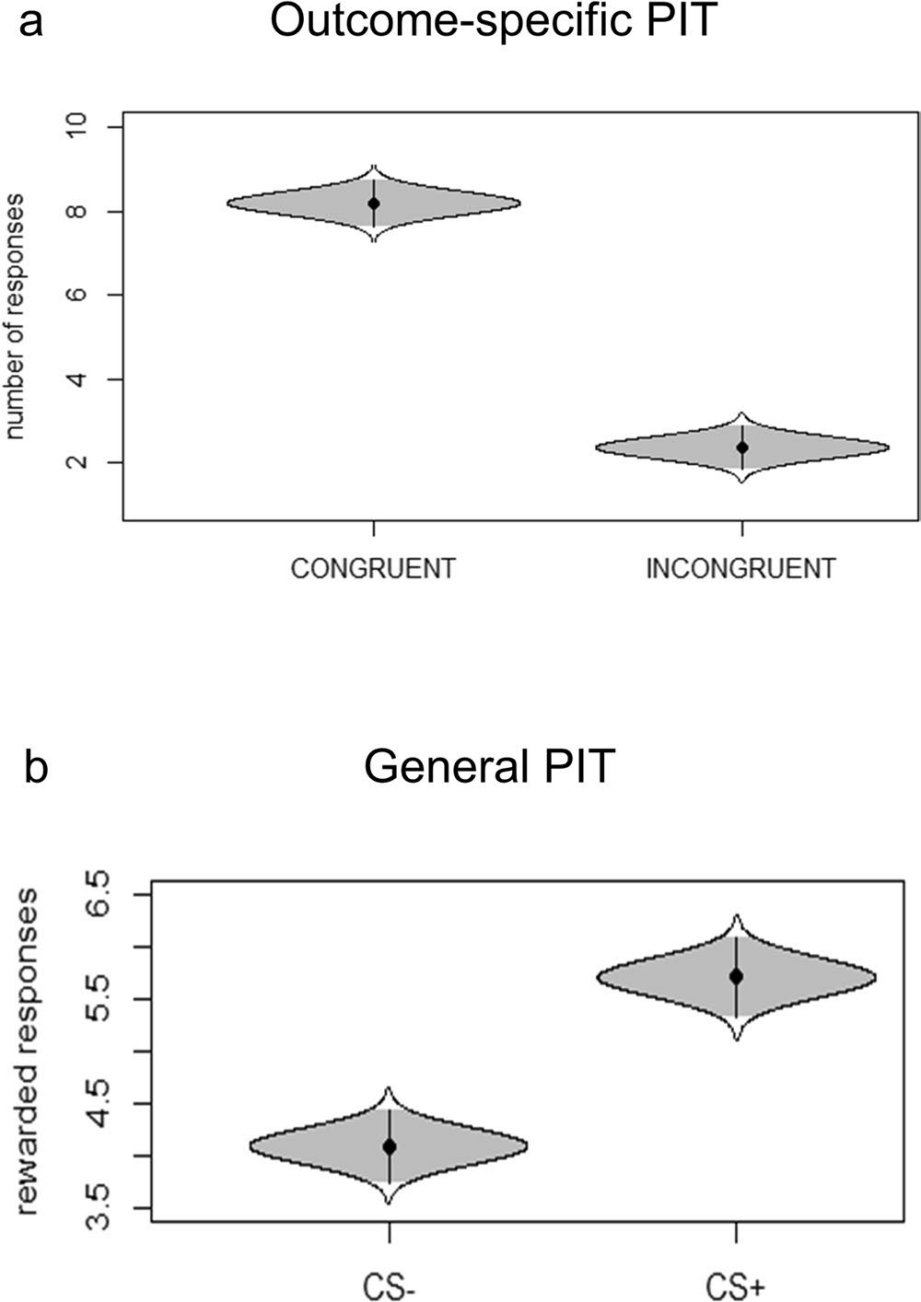
Cat’s eye plot of the Pavlovian-to-Instrumental Transfer. Panel (a) shows the outcome-specific PIT effect, as represented by a significant difference between the number of congruent (e.g., choosing R+1 when CS+1 is presented) and incongruent responses (e.g., choosing R+2 when CS+1 is presented). Panel (b) shows the general PIT effect, as represented by an increased propensity to choose the reward-associated response (R+) when a reward-associated stimulus is presented (CS+) as compared to the unrewarded stimulus is presented (CS−).

*The outcome-specific transfer* was tested considering only those trials in which CS+1 or CS+2 were presented while choosing between R+1 or R+2. The rationale of the outcome-specific transfer is to test if a CS (i.e., a reward-paired cue) is able to elicit a response independently associated with the same reinforcer. For this aim, all responses were categorized as congruent (choosing R+1 while CS+1 is presented, or choosing R+2 while CS+2 is presented) or incongruent (choosing R+1 while CS+2 is presented, or choosing R+2 while CS+1 is presented) and compared. Response (congruent/incongruent) served as the independent variable and number of responses as the dependent variable. Results showed a significant difference (F(1, 99) = 152; p < 0.0001; part. η^2^ = 0.61; BF_10_ = 1.15e + 37; err% = 1.74) between congruent (number of responses m = 8.28; sd = 2.64) and incongruent (number of responses m = 2.25; sd = 2.37) responses. Figure 3a shows means and clearly separate 95% confidence intervals. Form this results it is possible to conclude that participants showed outcome-specific transfer, as the task-irrelevant presence of a CS+ induced a preference for the instrumental response previously associated with the same reward.

#### General PIT

*The general transfer* was tested by comparing the preference for rewarded responses when presented with a CS+, relative to the CS−. CS (CS+/CS−) served as the independent variable and the number of rewarded responses (R+1 or R+2) as the dependent variable. Results showed a significant difference (F(1, 99) = 4.81; p = 0.03; part. η^2^ = 0.08; BF_10_ = 784841584730; err% = 1.11) between CS+ (m = 5.71; sd = 5.05) and CS− (m4 = 4–09; sd = 4–72). Figure 3b shows means and clearly separate 95% confidence intervals. From this result it is possible to conclude that participants showed general transfer, as the task-irrelevant presence of a CS+ induced a preference for the instrumental response previously associated with a (different) reward, as compared to the presence of the CS−.

### Working memory and indexes of learning

It was checked whether working memory could be related to the participant’s ability to learn during instrumental and Pavlovian learning tasks^32^. No significant correlation was found neither between working memory and instrumental learning index (t(98) = 0.81, p = 0.41, r = 0.08, 95% CI = −0.11 0.27; BF_10_ = 0.32) nor between working memory and Pavlovian learning index (t(98) = −0.34, p = 0.72, r = −0.03, 95% CI = −0.23 0.16; BF_10_ = 0.24).

### Working memory and PIT

**Figure 4:**
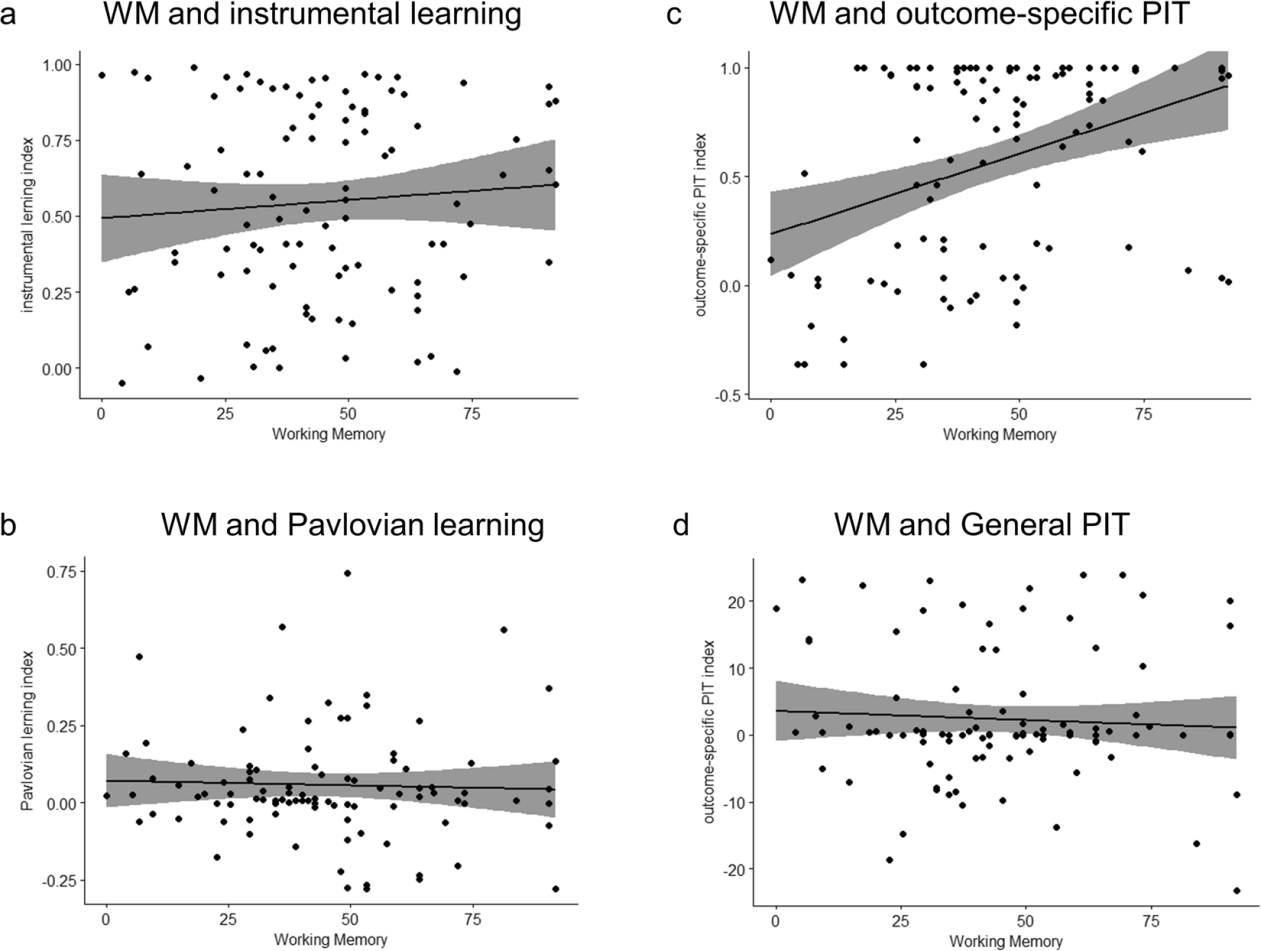
Correlation between Working Memory and Pavlovian-to-Instrumental Transfer. Panel (a) shows absence of correlation between working memory and instrumental learning index. Panel (b) shows absence of correlation between working memory and Pavlovian learning index. Panel (c) shows a significant positive correlation between working memory and outcome-specific PIT. Panel (d) shows absence of correlation between working memory and general PIT. The grey area represents the 95% confidence interval.

A first approach to investigate the relationship between working memory and PIT was to calculate two separate Pearson’s product-moment correlation coefficients for outcome-specific and general effect, respectively. Results showed a significant positive correlation (t(98) = 3.78, p = 0.0002, r = 0.35, 95% CI = 0.17 0.51; BF_10_ = 124.08) between working memory and outcome-specific transfer (Fig. 4c), whereas no correlation was found (t(98) = −0.59, p = 0.55, r = −0.06, 95% CI = −0.25 0.13; BF_10_ = 0.27) between working memory and general transfer (Fig. 4d).

### Working memory and outcome-specific PIT

**Figure 5:**
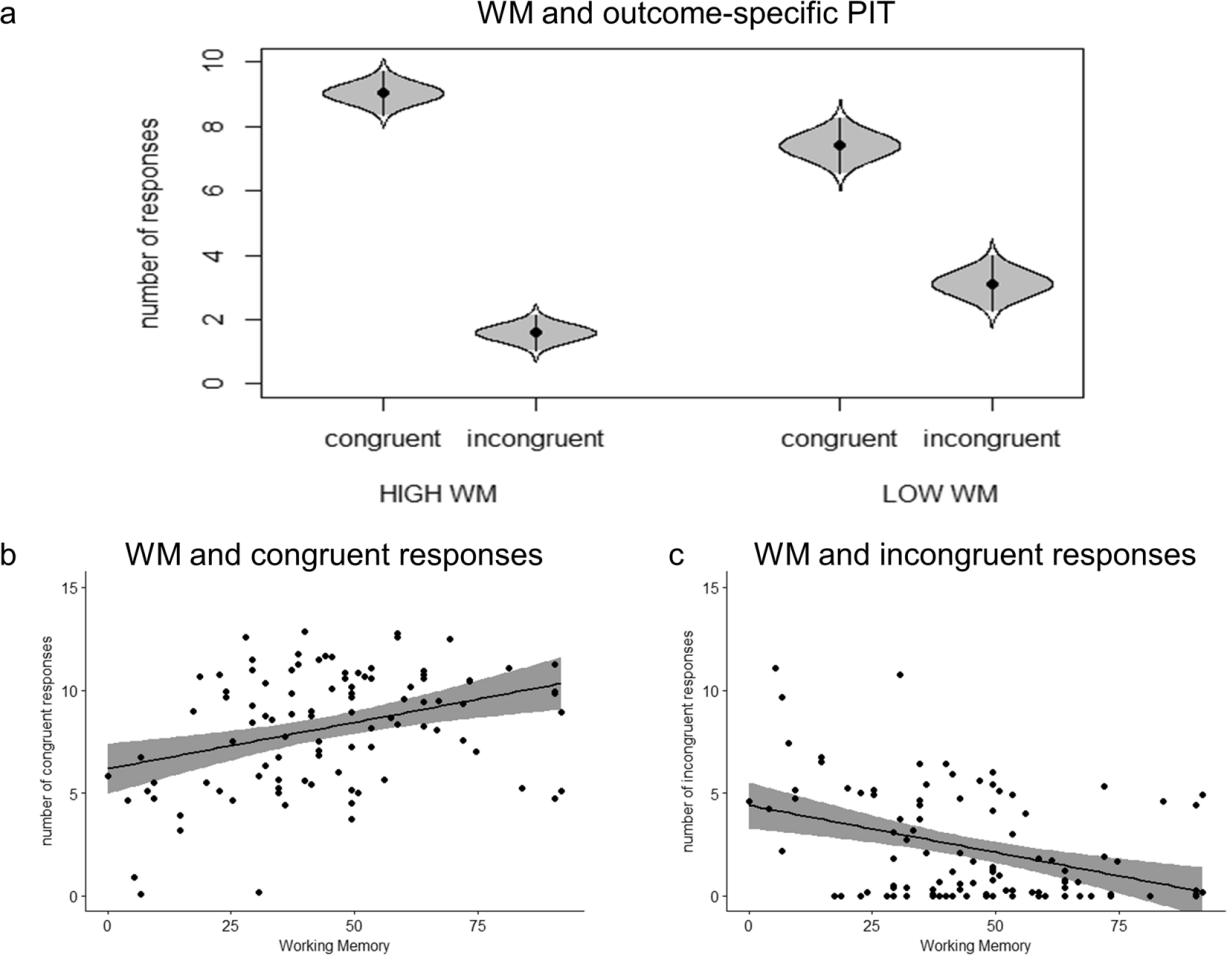
Working Memory and outcome-specific Pavlovian-to-Instrumental Transfer. Panel (a) shows the raw number of responses in the high and low working memory (WM) groups. Panel (b) shows positive correlation between working memory and the number of congruent responses. Panel (c) shows negative correlation between working memory and the number of incongruent responses. The grey area represents the 95% confidence interval.

To further inspect the relationship between individual differences in working memory and outcome-specific PIT, the raw number of congruent and incongruent responses was taken into account. The whole sample was divided into two groups with a median split, in order to separate individuals with high and low working memory working memory ability (median = 39). Working memory ability (high/low) and response (congruent/incongruent) served as the independent variable. The number of responses was used as the dependent variable. Results showed a significant difference (F(1, 98) = 165.14; p < 0.0001; part. η^2^ = 0.63; BF_10_ = 1.97e + 31; err% = 1) between congruent (m = 8.28; sd = 2.64) and incongruent (m = 2.25; sd = 2.37) responses. A significant interaction between working memory and response was also found (F(1, 98) = 8.62; p = 0.004; part. η^2^ = 0.08; BF_10_ = 8.36e + 32; err% = 2.77), where congruent responses were significantly higher in the high working memory group (m = 9.02; sd = 2.34) as compared to the low working memory (m = 7.56; sd = 2.74) group, and incongruent responses were significantly lower in the high working memory group (m = 1.58; sd = 1.96) as compared to the low working memory (m = 2.89; sd = 2.56) group (Fig. 5a).

Moreover, correlation analysis (Pearson’s product-moment) evidenced that working memory ability was not related to the overall number of responses performed during the PIT phase (t(198) = 0.05, p = 0.95, r = 0.003, 95% CI = −0.13 0.14; BF_10_ = 0.16), but positively correlated with the number of congruent responses (t(198) = 3.44, p = 0.0008, r = 0.32, 95% CI = 0.14 0.49; BF_10_ = 44.85) and negatively correlated with the number of incongruent responses (t(198) = −3.73, p = 0.0003, r = −0.35, 95% CI = −0.51 − 0.17; BF_10_ = 108.12) (Fig. 5b,c).

### The contribution of working memory and all learning indexes

**Figure 6:**
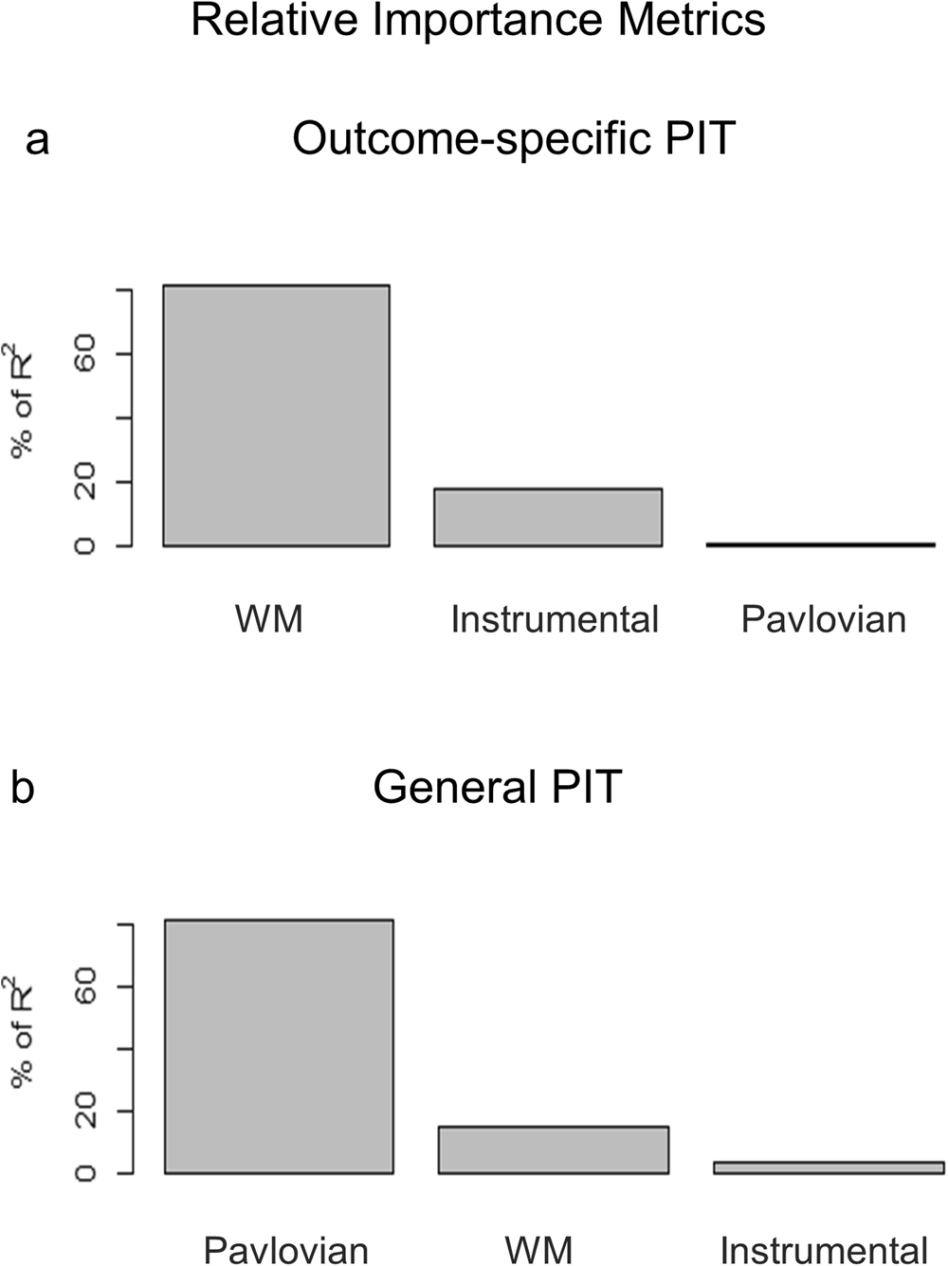
Contribution of working memory and learning factors. Relative importance metrics for outcome-specific (**a**) and general (**b**) PIT effect were calculated from two separate ordinary linear models. The PIT index (outcome-specific or general) was used as dependent variable and instrumental learning index, Pavlovian learning index, and working memory (WM) as independent factors. Metrics are expressed as a percentage of explained variability (R^2^) and factors are plotted in descending order.

To further evaluate the specific contribution of working memory as well as other learning factors to the expression of transfer, two ordinary linear models were fitted with either outcome-specific or general PIT indexes as dependent variables and instrumental learning index (number of responses), Pavlovian learning index (change in liking), and working memory as independent factors. The model on outcome-specific transfer was statistically significant (F(3,96) = 5.60, p = 0.001, R^2^ = 0.15). In particular, working memory proved to be the major determinant of the outcome-specific effect. The model on general transfer was not statistically significant (F(3,96) = 0674, p = 0.57, R^2^ = 0.02). All estimates and relative importance metrics are reported in Table 2 and Fig. 6.

## Discussion

The present study examined the relationship between individual differences in working memory capacity and two forms of Pavlovian-to-Instrumental Transfer (PIT), namely outcome-specific and general. For the first time, a dissociation between outcome-specific and general PIT was related to the role played by working memory in the mediation of this effect. More specifically, individual working memory was strongly correlated with outcome-specific PIT (Fig. 4c), but no relation with general PIT (Fig. 4d). Importantly, the data here presented suggest that this relation was not secondary to an influence of working memory on the acquisition of the learning contingencies. Indeed, neither instrumental nor Pavlovian learning indexes were related to working memory (Fig. 4a,b). This evidence is in line with the idea that, rather playing a role in the acquisition of conditioning^29^, individual differences in working memory may become crucial for the recall and strategic use of past learning. However, since the present experiment was not specifically designed to address this hypothesis, conclusion on this issue should be cautious^32^.

A deeper analysis of the relation between working memory and outcome-specific PIT, further highlighted that this connection was not related to an increase in the number of responses performed (no difference in the mean number of responses between high and low working memory groups), but rather to a precise imbalance between congruent and incongruent responses (Fig. 5a). In other words, even if the overall number of responses stays the same, people with higher working memory tend to be more selective in their choices (i.e., they make more congruent responses and less incongruent responses). This interpretation was confirmed by the positive correlation found between individual working memory ability and the number of congruent responses (Fig. 5b) and by the negative correlation between individual working memory ability and the number of incongruent responses (Fig. 5c).

Finally, when looking at the specific contributors to the transfer effect, it was further confirmed that an explicit representation of the instrumental contingencies and individual working memory ability were the strongest determinants of outcome-specific PIT (Fig. 6a). This result particularly supports the hypothesis that higher working memory is associated with higher outcome-specific PIT effect because a detailed representation of all outcomes and response-outcome pairings is at the foundation of this particular form of transfer (S− > O− > R chain). Although for general PIT the model was not statistically significant, it may be interesting to note that the strongest contributor was represented by an implicit measure of Pavlovian conditioning (change in CS liking) (Fig. 6b).

Taken together, the present findings support the idea, also highlighted in previous works^20,33^, that outcome-specific and general PIT are sustained by different underlying mechanisms. More precisely, working memory seems to be less relevant when the Pavlovian cue informs the easy choice between two responses, of which only one was previously associated with a reward (general PIT). In such a situation, only action values need to be considered. Conversely, working memory becomes a crucial requirement when the same information from the same Pavlovian cue can guide a more difficult choice between two actions with similar motivational values (outcome-specific PIT). In this case, a precise integration between the specific nature of the outcome and the consequences associated with each response is required. In other words, if a task-irrelevant cue signals a potentially useful information for a complex choice, the working memory comes into play to integrate past experiences about R → O and S → O associations in order to guide such choice^18,23,24^. Importantly, such influence can have adaptive or maladaptive implications^12–18^.

Given the current understanding of the PIT phenomenon and its implications in both daily life and clinical conditions (see introduction), it seems important to explore the extent to which Pavlovian stimuli can either be a strategic guide for behavior or exert a maladaptive control over instrumental responding. An attempt will be made to interpret the present findings in this sense.

When interpreting a phenomenon like the PIT effect, a mediating effect of the outcome representation - whether its value or its sensory features - can hardly be doubted^34^, but the nature of this link is still unclear. Many pieces of evidence point to the fact that the working memory system is critically involved in the guidance of action selection through executive functions, and that cognitively controlled decision-making relies on working memory^21,35,36^. Many findings also suggest that individual differences in working memory play no role in performances depending on automatic processes^37^. In this regard, it would be interesting to test whether a differentiation between more implicit and executive components of working memory (i.e., the phonological loop and the central executive) may further clarify its role in outcome-specific and general PIT^23,38^.

Following this narrative and the demonstrated relation with individual working memory capacity, can it be argued that outcome-specific PIT is guided by an explicit anticipation of the associative rules, thus being cognitive and strategic? Likewise, can the absence of a relation between working memory and general PIT be interpreted as evidence that more automatic processes are responsible for this form of PIT? Although a number of studies go along with both speculations^12,15,18,30,34,39–45^, evidence is still indirect and careful consideration should be taken in drawing such conclusions. In the present study, for example, a limitation is represented by the presence of a smaller effect size for the general PIT (part. η^2^ = 0.14), as compared to the specific PIT (part. η^2^ = 0.61), which may weaken the possibility of uncovering a relation with the variables included in the subsequent analysis, including working memory.

Above all, the critical question is whether individual differences in working memory can be reflected in different facets of cue-guided behaviors and, therefore, in a different propensity to develop maladaptive behaviors. Can higher working memory be an advantage in guiding behavior? We propose that the critical role played by working memory resides in the integration of the values coming from two different sources: the cue and the response^46,47^. In outcome-specific PIT, multiple sources of information converge to signal a higher chance to find a reward (congruent response). The role of working memory should not be intended here as a mere cognitive resource^35^, but rather as the mechanism defining the best choice. The data here presented may indicate that an adaptive component of PIT, as reflected by the outcome-specific form, may go along with working memory to flexibly guide behavior toward the most convenient choice. If that is the case, higher working memory ability could constitute a competitive advantage, whereas a lack of working memory could be a potential risk factor towards the development of maladaptive conducts. Within this scenario, a clear distinction between the two mechanisms appears important for clinical purposes, as more automatic responses may be more prone to evolve in a maladaptive behavior (like drug-seeking), as compared to strategically guided actions.

Drug addiction has been conceptualized as the endpoint of a transition from voluntary, through habitual and ultimately compulsive drug use, which directly depends on interactions between Pavlovian and instrumental learning processes^16,48^. Drug-related forms of PIT have been clearly reported in the literature^12,13,15,49,50^, but there is no clear evidence about the prevalence of one form over the other (outcome-specific vs. general) in the addicted population. A possibility is that while occasional use of drugs can be initially guided by an outcome-specific PIT-like mechanism, while the endpoint to a compulsive use can be more governed by a general PIT-like mechanism. Such a distinction may have profound implications in the treatment and prevention of addiction. A propensity to present one or the other form of transfer could be intended as a marker of vulnerability to cue-induced pathological behaviors. Importantly, these implications could be not limited to addiction, but extended to compulsive behavior and neuropsychiatric disorders, like depression or schizophrenia^16–18^.

The view proposed here and elsewhere^20,33^ is consistent with the idea that, at least in humans, outcome-specific and general PIT are sustained by a different cognitive mechanism, potentially involving different paths of the underlying corticostriatal loop^9,16,18,51,52^. A major hypothesis concerning the mechanisms behind the transition from adaptive to maladaptive behavior proposed that the initial cortical control performed by the prefrontal cortex can gradually evolve in a more striatal - ventral to dorsal - control of behavior^16,48^. However, more evidence is needed about the presence of distinct neural markers of outcome-specific and general PIT in humans. Furthermore, an additional question is whether the two separate systems cooperate to reach optimal behavior or are in competition for action control. Importantly, if confirmed by converging evidence, such differentiation could have major implications for understanding the determinants of adaptive and maladaptive behaviors.

## Materials and Methods

### Participants

One-hundred volunteers (50 female; mean age = 22.9 years, sd = 2.45; mean education = 15.1 years, sd = 1.92) with no history of neurological diseases were recruited from the student population at the University of Bologna. The number of participants was determined based on a power analysis with the following parameters: effect size = 0.35; significance level = 0.05, power = 0.95. The effect-size was estimated based on previous independent literature reporting a relationship between working memory and reward processing^53–56^. All participants gave written informed consent to take part in the experiment. The study was conducted in accordance with institutional guidelines and the 1964 Declaration of Helsinki. It was approved by the Ethics Committee for Psychological Research of the University of Bologna.

### Stimuli

Five black squares (4 cm^2^) were displayed on a 17-inch color monitor with a black background. The squares were highlighted by a white frame (2 mm thickness) and positioned as follows: upper center, bottom center, right center, left center and center of the screen. Three fractal images (balanced for luminance, complexity and color saturation) were used as Pavlovian conditioned stimuli (CS) and presented within the top center square. Two images of food items, surrounded by a light-yellow ring (equally sized), were used as separate rewards and a white ‘x’ was used as a neutral outcome (no-reward). The same visual environment was used in all phases of the PIT task.

Rewards were individually tailored for each participant. Before the experimental session, every participant was required to rate the subjective liking and wanting of 12 savory foods and 12 sweet foods using two separate 10-items Likert scales. Ratings ranged from 0 (not at all) to 10 (very much). Foods were presented using the same images subsequently implemented in the PIT task. Based on the subjective preference expressed, one savory food and one sweet food with similar liking and wanting ratings were selected and used as rewards in the subsequent learning task.

### Procedure

The whole experiment consisted of three reinforcement learning tasks – used to assess PIT - and one working memory task – used to assess working memory. On arrival, participants were comfortably seated in a silent room and their position was centered relative to the screen, at a viewing distance of 50 cm from the screen.

#### Pavlovian-to-Instrumental Transfer (PIT) Task

PIT was assessed following a standard paradigm consisting of three phases, which mirrored the structure used in previous studies^11^: (1) Instrumental Conditioning task, in which participants learned a response-contingent reward; (2) Pavlovian Conditioning task, in which participants learned a cue-contingent reward; (3) PIT task, during which the influence of task-irrelevant Pavlovian cues on instrumental responding was tested. Participants were aware that, at the end of the experiment, they would be recompensated with an amount of food proportional to the number of food pictures (to be considered as points) visualized during all tasks. The detailed composition of trials for all task phases is reported in Table 1. In each task, participants were required to pay attention to the screen and follow the instructions reported at the beginning of the task. A few example trials were always performed. A computer running OpenSesame software^57^ controlled stimulus presentation.

**Table 1:** Trial composition for all task phases.

TRIALS		CONTINGENCY		
Instrumental Conditioning		R+1 → O1 R+2 → O2 R− → NR		
Pavlovian Conditioning		CS+1 → O1 CS+2 → O2 CS− → NR		
PIT	Outcome-specific	CS+1: R1 vs R2 CS+2: R1 vs R2		
	General	CS+1: R2 vs R− CS+2: R1 vs R−	vs	CS−: R2 vs R− CS−: R1 vs R−

**Table 2:** Contribution of working memory and learning factors to the PIT effect.

	β	Std. Error	t	p	RI
**Outcome-specific PIT**					
Working Memory	0.01	0.01	3.61	<0.001	0.82
Instrumental	0.21	0.13	1.56	0.12	0.17
Pavlovian	−0.01	0.02	−0.21	0.82	0.01
**General PIT**					
Pavlovian	0.60	0.48	1.24	0.21	0.81
Working Memory	−0.02	0.04	−0.50	0.61	0.15
Instrumental	0–84	3.14	0.26	0.79	0.03

The table reports estimates of the linear model and relative importance metrics (RI).

#### Instrumental conditioning phase

Participants were required to learn the association of three possible responses (three white squares horizontally centered on the screen) with their respective reward. Two responses were paired with two different rewards (R+1 and R+2), while the other was paired with a neutral outcome (R−). The task followed a partial reinforcement schedule, such that when a specific response was rewarded, the same response would not be rewarded for the following 2 to 4 seconds. In each trial, only two out of the three squares were presented in white and indicated as possible responses to be selected with a mouse click. After each response, a corresponding neutral or reward outcome appeared for 1 second in the bottom square (Fig. 1a). A fixation cross with a jittered duration ranging between 1 and 1.5 seconds was used as intertrial interval. Response options were not available during this period. The rationale of this task was to learn the association between a specific instrumental response and its corresponding outcome. The response-outcome association was counterbalanced across subjects. The task consisted of a total of 27 trials (9 trials for each pair of responses), each lasting 10 seconds, during which participants were free to perform as many responses (i.e., mouse clicks) as they wished. The task lasted about 6 minutes.

#### Pavlovian conditioning phase

In each trial, one of three possible visual cues (fractal images) appeared for 5 seconds within the upper square, followed by a white patch within the bottom square. Participants were instructed to press the left-Ctrl button on the computer keyboard, as quickly as possible, to remove the patch and discover the outcome hidden behind it. The outcome was presented for 1 second. A fixation cross with a jittered duration ranging between 1 and 1.5 seconds was used as intertrial interval. Stimuli were not presented during this period. Two of the visual cues were associated with a reward (food picture) on 80% of trials (CS+1 and CS+2), while the other cue was associated with no reward (neutral cue) on all trials (CS−) (Fig. 1b). The stimulus-outcome association was counterbalanced across subjects. The speeded key press aimed to remove the patch and release the hidden reward has been used in previous studies^11,52,58,59^ as a measure of conditioning that mirrors the reward collection upon presentation of the CS+, observed in animal studies^9,30,60^. To avoid a possible instrumental component, participants were told that, in this phase, the reward was not dependent upon their response and that, after 3 seconds, the patch hiding it would disappear even without any key press. This was explicitly demonstrated during the example trials. Crucially, responses in the instrumental and Pavlovian tasks were orthogonalized such that mouse-clicks in two spatially different locations served as instrumental response, while a unique key-press on the left ctrl button was used here to remove the patch. The rationale was to observe faster reaction times when a reward was predicted (CS+ condition), as compared to when a neutral outcome was predicted (CS− condition). The task consisted of 75 trials (25 per each visual cue), for a total duration of about 7 minutes.

#### Transfer phase

Participants were given the same instructions used for instrumental conditioning, i.e. they were required to perform a choice between the two squares available in each trial. The task was identical to the instrumental conditioning phase, except that (a) while performing choices, task-irrelevant Pavlovian CSs were presented randomly within the upper square and (b) the whole task was performed under extinction, so no reward was ever delivered (Fig. 1c). Extinction is a standard procedure for assessing PIT effect, both in human and animal research, since it allows to test the influence of Pavlovian cues on instrumental responding without the confounding effects of the reward^52,61–63^. The global rationale of this task was to test the ability of a Pavlovian cue to drive an instrumental response previously associated with a reward. Two different trials types were designed to disentangle outcome-specific and general transfer (Table 1, PIT). In outcome-specific trials, participants were presented with a task-irrelevant CS+ (CS+1 or CS+2) while required to choose between two available response options (R+1 and R+2) previously paired with two different reward outcomes, of which only one was also previously paired with the concurrently presented CS. So, for example, if the CS+1 was presented, choosing R+1 would constitute a congruent response, while choosing R+2 would constitute an incongruent response. Similarly, if the CS+2 was presented, choosing R+1 would constitute an incongruent response, while choosing R+2 would constitute a congruent response. Evidence for outcome-specific PIT would be seen if the presence of the CS induced a higher rate of congruent responses, as compared to incongruent responses. In general trials, participants were presented with a task-irrelevant CS (either reward-associated or neutral) and required to choose between two available response options none of which was compatible with the CS currently available (i.e., the responses were always associated with a different or no outcome). So, if the CS+1 was presented the available response options were R+2 and R−; if the CS+2 was presented the available response options were R+1 and R−; if the CS− was presented the available response options could be either R+1 and R− or R+2 and R−. Evidence for general PIT would be seen if participants favoured the R+ during the presentation of a CS+, as compared to the CS−. The task consisted of 36 trials of 10 seconds (6 trials for each association of cue and response pair), during which subjects were free to perform as many responses (i.e., mouse clicks) as they wished. The task lasted for about 7 minutes.

CS liking: As a further measure of learning, the visual cues used as CSs were presented before and after Pavlovian conditioning. Participants were required to rate their subjective liking on a 9-items Likert scale. The rationale was to observe an increase in liking scores for CS+1 and CS+2, after Pavlovian conditioning, but not for the CS−.

Explicit learning: At the end of the two conditioning tasks, participants were presented with all instrumental options and visual cues and were required to associate them with their corresponding outcome.

#### Working memory

Working memory was evaluated with the automated operational span task (AOSPAN), mirroring the same procedure extensively described elsewhere^64^. AOSPAN requires participants to solve a series of mathematical operations while trying to remember a set of unrelated letters. Participants were presented with one mathematical operation to solve and one letter to remember at a time. The practice session for this task was divided into three sections. The first practice section was letter span: a letter appeared on the screen, and participants were required to recall the letters in the same order in which they were presented. The recall was performed by clicking the box next to the appropriate letter in the correct order. This phase was untimed. Following this, the computer provided feedback about the number of letters correctly recalled in the current set. In the second practice session, participants practiced the mathematical operations of the task and were instructed to solve the operation as quickly as possible. After each operation, participants were given feedback on their accuracy. This practice session served to familiarise them with the mathematical session of the task as well as to calculate how long it would take each person to solve the mathematical operations, in order to account for individual differences. Afterward, the program estimated the subjective mean time required to solve the equations. This time (plus 2.5 standard deviations) was then used as a time limit for the mathematical operations of the experimental session for that individual. In the final practice session, participants performed both the letter recall and mathematical operations together, just as they would do in the real task. Finally, the program progressed to the real trials, which consisted of three sets of 3 to 4 alternations of operation and letter, for a total of 75 letters and 75 mathematical problems. The order of set size was random so that participants could not predict the number of items. In all conditions, letters remained onscreen for 800 ms. The task took approximately 20 min to complete. The score obtained was calculated as the percentage of trials correctly performed in the real task phase only.

### Statistical analysis

All statistical analyses are performed using R software v3.3.2 (R Core Team, 2016) and RStudio v1.0.136 (RStudio Team, 2016). The following packages were implemented: lme4, afex, stats, lsmeans, relaimpo, plotmeans, multicon, BayesFactor. In linear mixed-effects models, subjects were always modelled as a random effect. All post-hoc analysis are Bonferroni-corrected. Reported p-values are always intended as two-tailed. The Bayes Factor is reported for all analysis as the probability associated with the alternative hypothesis over the null hypothesis (BF_10_). The default number of Monte Carlo samples was always 10000. The estimated proportional error (err%) associated with the Bayes Factor is reported only of higher than 0. In the text, values are reported as mean (m) and standard deviation (sd). In all figures, data are reported as cat’s-eye pictures with a 95% confidence interval^65^. The curves represent the sampling distribution and are centered on the sample mean. The grey indicates 95% of the area between the curves. The horizontal width of each cat’s-eye picture represents the relative likelihood of the interval. Assumptions for a correct use of parametric statistics are always assessed. For the linear model fit and relative importance calculation, the absence of correlation between all regressors was checked. Relative importance was assessed with lmg method to quantify the contribution of each individual regressor to the model^66^. Relative importance estimates the proportionate contribution of each regressor to the full model’s explained variance (expressed as R^2^), considering both its direct and its indirect (i.e., combined with the other regressors) influence in the regression equation^66^. Relative importance metrics were normalized to sum 100 and all indices introduced in the model were rescaled from 0 to 100.

### Learning and transfer indexes

To further conduct correlation and regression analyses, performance indexes were calculated for each participant in each task.

*Instrumental learning index* was calculated by applying the subsequent formula to the number of responses performed: [(reward-associated − unrewarded)/total]. A positive score corresponded to a preference for reward-associated (R+1 or R+2) over unrewarded (R−) responses, and vice-versa.

*Pavlovian learning* was characterized by two measures, reaction times and CS liking. To create a Pavlovian learning index, we selected the measure that reported a stronger effect-size, namely CS liking (part. η2 = 0.34). The index was created by applying the following formula to the ratings: [(CS+post − CS+pre) − (CS−post − CS−pre)]. A positive score corresponded to a stronger change in liking in the CS+ condition, as compared to the CS− condition, and vice-versa.

*Outcome-specific transfer index* was calculated by applying the subsequent formula to the number of responses performed: [(congruent − incongruent)/total]. A positive score corresponded to a preference for congruent over incongruent responses, and vice-versa.

*General transfer index* was calculated by applying the subsequent formula to the preference index described above: CS+ − CS−. A positive score corresponded to a preference for reward-associated responses (R+1 or R+2) when presented with a reward-associated cue (CS+1 or CS+2), as compared to the unrewarded cue (CS−), and vice-versa. Please note that the division for the total number of responses was not applied in this case because this index was obtained from the preference index, which was already corrected for inter-individual variability, by dividing for the total number of performed responses. A further correction would then result in a misleading score.

## Supplementary information

Supplementary analysis
