# Supplementary material for: Individual differences in working memory capacity and cue-guided behavior in humans
Source: Sci Rep. 2019 May 13;9:7327. doi: 10.1038/s41598-019-43860-w (PMC6514037; doi:10.1038/s41598-019-43860-w)
Supplement: Supplementary file 1 — Supplementary analysis [file 41598_2019_43860_MOESM1_ESM.docx]

**Individual differences in working memory capacity and cue-guided behavior in humans**

Sara Garofalo, Simone Battaglia & Giuseppe di Pellegrino

# Additional Results

## Instrumental Conditioning

*Implicit learning.* The instrumental conditioning learning index was tested against 0. This analysis (t(99)= 17.191; p<.0001; CI= 0.48 0.61) confirmed a predominance of reward-related responses as compared to non-rewarded responses (mean = 0.56, sd= 0.32).

## Pavlovian Conditioning

*Implicit learning.* CS (CS+_1_/CS+_2_/CS-) served as the independent variable and change in CS liking (rating post – rating pre) as the dependent variable. Results showed a significant main effect of CS (F(1.82, 180.47)=43.65; p <.0001; part. η^2^=.31; BF_10_=305.35; err%=0.76). Post-hoc analysis confirmed a statistically significant difference (ps <.001) between CS+_1_ (m=0.59; sd=1.33) and CS- (m=-.93; sd=1.50), as well as between CS+_2_ (m=.97; sd=1.59) and CS-, but not between CS+_1_ and CS+_2_ (p=.1). Figure 1S shows means and clearly separate 95% confidence intervals. These results indicated that, after undergoing Pavlovian conditioning, participants significantly increased their liking for reward-paired cues (CS+_1_ and CS+_2_), as compared to the neutral cue (CS-), thus confirming effective Pavlovian conditioning.


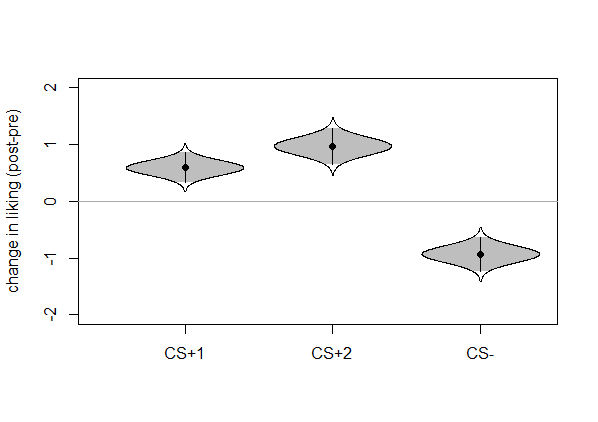


**Figure 1S - Pavlovian learning**

Change in liking (post-pre) of the conditioned stimuli (CSs), where an increase was observed for the reward-associated stimuli (CS+1, CS+2) and a reduction for the unrewarded stimulus (CS-) was observed.

## Pavlovian-to-Instrumental Transfer (PIT)

***Outcome-specific PIT***

*The* outcome-specific PIT index was tested against 0. This analysis (t(99)= 12.52; p<.0001; CI= 0.47 0.67; BF_10_=1.14e+37) confirmed a prevalence of reward-related responses as compared to non-rewarded responses (mean = 0.57, ds= 0.31).

***General PIT***

The general transfer was tested by comparing the preference for rewarded over unrewarded responses when presented with a CS+, relative to the CS-. For this purpose, a preference index was created by subtracting, for each trial, the number of R- from the number of R+ choices and dividing for the total number of responses [(reward associated - unrewarded) / total]. Thus, a positive score corresponded to a preference for R+ over R- during that trial, and vice-versa. The resulting value was then compared between CS+ (either 1 or 2) and CS- trials. CS (CS+/CS-) served as the independent variable and preference index as the dependent variable. Results showed a significant difference (F(1, 99)=16.54; p<.001; part. η2=.14; BF_10_=281.98; err%=1.05) between CS+ (m=2.83; sd=7.81) and CS- (m=-1.15; sd=8.29). From this result, it is possible to conclude that participants showed general transfer, as the task-irrelevant presence of a CS+ induced a preference for the instrumental response previously associated with a (different) reward, as compared to the presence of the CS- (Fig.2S).


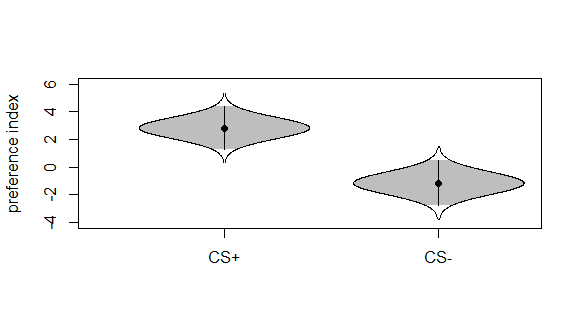


**Figure 2S – General PIT**

General PIT effect is represented by a higher preference index (propensity to choose an R+ over R-) when a reward associated stimulus is presented (CS+) as compared to the unrewarded stimulus is presented (CS-).
